# Prevalence of drug use, alcohol consumption, cigarette smoking and measure of socioeconomic-related inequalities of drug use among Iranian people: findings from a national survey

**DOI:** 10.1186/s13011-020-00279-1

**Published:** 2020-06-05

**Authors:** Mehdi Moradinazar, Farid Najafi, Farzad Jalilian, Yahya Pasdar, Behrooz Hamzeh, Ebraim Shakiba, Mohammad Hajizadeh, Ali Akbar Haghdoost, Reza Malekzadeh, Hossein Poustchi, Marzeyeh Nasiri, Hassan Okati-Aliabad, Majid Saeedi, Fariborz Mansour-Ghanaei, Sara Farhang, Ali Reza Safarpour, Najmeh Maharlouei, Mojtaba Farjam, Saeed Amini, Mahin Amini, Ali Mohammadi, Mehdi Mirzaei-Alavijeh

**Affiliations:** 1 Research Center for Environmental Determinants of Health (RCEDH), Health Institute, Kermanshah University of Medical Sciences, Kermanshah, Iran; 2 Social Development and Health Promotion Research Center, Health Institute, Kermanshah University of Medical Sciences, Kermanshah, Iran; 3 School of Health Administration, Faculty of Health, Dalhousie University, Halifax, Canada; 4 Modeling in Health Research Center, Institute for Future Studies in Health, Kerman University of Medical Sciences, Kerman, Iran; 5 Digestive Diseases Research Center, Digestive Diseases Research Institute, Tehran University of Medical Sciences, Tehran, Iran; 6 Digestive Oncology Research Center, Digestive Diseases Research Institute, Tehran University of Medical Sciences, Tehran, Iran; 7 Modelling in health Research Center, Shahrekord University of Medical Sciences, Shahrekord, Iran; 8 Health Promotion Research Center, Zahedan University of Medical Sciences, Zahedan, Iran; 9 Department of Pharmaceutics, School of Pharmacy, Mazandaran University of Medical Sciences, Sari, Iran; 10 Gastrointestinal and Liver Diseases Research Center, Guilan University of Medical Sciences, Rasht, Iran; 11 Liver and gastrointestinal Diseases Research center, Tabriz University of Medical sciences, Tabriz, Iran; 12 Gastroenterohe Pathology Research Center, Shiraz University of Medical Sciences, Shiraz, Iran; 13 Health Policy Research Center, Shiraz University of Medical Sciences, Shiraz, Iran; 14 Non-communicable diseases Research Center, Fasa University of Medical Sciences, Fasa, Iran; 15 Health Services Management, Arak University of Medical Sciences, Arak, Iran

**Keywords:** Inequalities, Drug use, Socioeconomic status, Concentration index, Iran

## Abstract

**Background:**

Drug use can lead to several psychological, medical and social complications. The current study aimed to measure and decomposes socioeconomic**-**related inequalities in drug use among adults in Iran.

**Methods:**

This was a cross-sectional study The PERSIAN Cohort is the largest and most important cohort among 18 distinct areas of Iran. This study was conducted on 130,570 adults 35 years and older. A structured questionnaire was applied to collect data. The concentration index (C) was used to quantify and decompose socioeconomic inequalities in drug use.

**Results:**

The prevalence experience of drug use was 11.9%. The estimated C for drug use was − 0.021. The corresponding value of the C for women and men were − 0.171 and − 0.134, respectively. The negative values of the C suggest that drug use is more concentrated among the population with low socioeconomic status in Iran (*p* < 0.001). For women, socioeconomic status (SES) (26.37%), province residence (− 22.38%) and age (9.76%) had the most significant contribution to socioeconomic inequality in drug use, respectively. For men, SES (80.04%), smoking (32.04%) and alcohol consumption (− 12.37%) were the main contributors to socioeconomic inequality in drug use.

**Conclusions:**

Our study indicated that drug use prevention programs in Iran should focus on socioeconomically disadvantaged population. Our finding could be useful for health policy maker to design and implement effective preventative programs to protect Iranian population against the drug use.

## Background

Drug use (refers to any scope of use of illegal drugs: heroin, amphetamines, barbiturates, cannabis, cocaine, hallucinogens, and opioids) is one of the major health, psychosocial and socioeconomic problems in the world, and can lead to several problems and complications for addicts, their families and society [1, 2]. There exists sex differences in the drug use in the communities; the rate of drug use in men has been significantly higher than women even though this sex gap has been steadily decreasing [3].

Iran is one of the countries where drug use prevalence has increased in recent years;this is due to various reasons such as shares border with Afghanistan in the Eastern of Iran which is the largest producer country of opium in the world, and a major route for substance transport to Europe [4]. Based on the World Health Organization (WHO), Iran has the highest rate of opium abusers in the world, and opium use in Iran is three times the global average [5]. The statistics show that there are about 2 million people use illicit drugs at a daily basis in Iran, which is about 2.7% of the population [6].

Providing knowledge about the determinants influencing drug use in the society can enable health policymakers to the development and the implementation evidence-based preemptive programs [7]. In the area of addiction tendency, various hypotheses have been expressed that none of them alone can explain this problem [8, 9]. Several studies have indicated the predictive nature of socioeconomic status (SES) against drug use such as alcohol consumption, opium, cigarette smoking, and cannabis; however, the association between SES and drug use is complex [10–14]. For example, the prevalence of cigarette smoking is higher among groups with low SES and they maybe more exposure to harms related cigarette smoking [10]. A higher addiction rate also found among individuals with low SES [12]. Contrary to these results, some studies [11] reported higher alcohol consumption and cannabis use among high SES.

Although studies have been conducted on the relationship between SES and drug use in Iran, these studies have used a small sample size with a focus on only one sex (especially men) [15–17]. Thus, more studies are required to predict socioeconomic-related inequalities in drug use among adults in Iran. Given the widespread incongruities among the findings of researches, our study focused on socioeconomic-related inequality to the drug use in among Iranian adult’s population using a large sample size obtained from the Prospective Epidemiologic Research Study (PERSIAN Cohort).

## Methods

### Study setting

The PERSIAN Cohort is the largest and most important cohort among 18 distinct areas of Iran. This study launched nationwide by the Ministry of Health and Medical Education (MoHME) in Iran to provide information about Non-communicable Diseases (NCDs) among Iranian adults (aged 35 and above). The PERSIAN Cohort Study is a prospective study with purpose to include 180,000 Iranians aged 35–70 years from 18 geographically distinct areas of Iran. While the MoHME oversees the project, researchers at local Iranian medical universities carry it out. The cohort has started in 2014 and collects information between 5000 and 10,000 people from all Iranian ethnic groups in each district area. The financial support for the study is provided by the MoHME, and the deputy of research in the medical universities in the 18 distinct areas of Iran. The protocol of PERSIAN Cohort study (including: objectives, outcomes of interest, design of study, site selection, participant selection, sample size, sampling methods, inclusion criteria, and quality assurance and quality control was published in American Journal of Epidemiology [18] and Archives of Iranian Medicine [19]. The Iranian people comprise individuals of many ethnicities. Appendix 1 shows the characteristics of cohort sites in Iran.

### Study tools

The cohort questionnaire is administered by trained interviewers.

#### Physical activity

Physical activity was measured weekly based on all the physical activity related to exercise, work, and recreation in the past 24 h. Physical activity questionnaire includes 19 light, moderate, and severe activities. MET is the amount of oxygen consumed at rest (about 3.5 ml 02/kg/min) and equals to resting metabolic rate. Physical activity levels were classified as low (24–36.5 MET-hours per week), moderate (MET-36.6-44.9 h per week) and heavy (MET- ≥ 45 h per week) [18].

#### Drug use variable

To assess whether or not the subjects had a history of drug use (included: heroin, amphetamines, barbiturates, cannabis, cocaine, hallucinogens, and Opioids), a question was asked: “Have you used illicit drug more than one time during a lifetime?” The reply options for each drug question was “Yes” or “No”.

#### Cigarettes smoking

Smoking status evaluated based the one National Health Interview Survey (NHIS). If respondents smoked ≥100 cigarettes in their lifetime, they were defined as smokers. The ex-smoker refers to an individual who has given up a cigarette smoking. Former smokers were previous smokers but are no longer smoking [20].

#### Water pipe smoking

To assess whether or not the participants had experienced water pipe smoking, we used one questions “Have you ever water pipe smoking at during a lifetime?” which the response category was yes or no.

#### Alcohol consumption

To assess whether or not the respondents had a history of alcohol consumption a question was asked: “Have you consumed alcoholic drinks more than one time during a lifetime?” The reply options for alcohol consumption question was “Yes” or “No”.

#### Socioeconomic status index

A principal component analysis (PCA) method was used to construct an SES index of respondents in the Cohort study. Available information on, infrastructure facilities (source of drinking water, sanitation facility), housing condition (e.g. the number of rooms, type of home ownership) and ownership of a range of durable assets (e.g. dishwasher, car, television), and education level in the dataset was used in the construction of SES variable for each participants. Participants of the study categorized into five SES quintiles, from the lowest (1st quintile) to the highest (5th quintile) SES groups.

#### Measuring socioeconomic-related inequality in drug use

Socioeconomic-related inequality in drug use was estimated using the Concentration index (C) approach and the Concentration curve [19]. The concentration curve is a two-dimensional graph. The horizontal axis shows the cumulative percentage of the population ranked from the poorest to the richest, and the vertical axis indicates the cumulative percentage of the health variable (in the present study: drug use). The 45-degree line represents full equality in the distribution of drug use. If the drug abuse rate is higher among socioeconomically disadvantaged individuals, the concentration curve places above the equality line. The C is extracted from the Concentration curve and equals twice the space between the Concentration curve and the equality line (45-degree). If the index is zero, this means that the variable is distributed equally among SES groups. The following formula was used to measured inequality and estimated the C (equation (1):

1 $$ \mathrm{C}=\frac{2\ast \mathit{\operatorname{cov}}\left({y}_i{r}_i\right)\ }{\mu } $$

Where *y*_*i*_ is health variable for the person *i*, *μ* it’s mean of the health outcome variable and *r*_*i*_ the fractional rank. Considering that the drug use in our study was a binary variable, the minimum and maximum values of the C does range between − 1 and + 1. Thus, the C was normalized by dividing the estimated value of the concentration index by 1 − *μ* (equation (2) [20].

2 $$ {C}_n=\frac{C_I}{1-\mu } $$

To determine the contribution of each factor to socioeconomic inequality in drug use, we decompose the estimated *C*_*n*_ (equation (3) [21]. If we have a regression model relating a set of demographic (age, sex, marital status), behavioural (cigarette smoking, water pipe smoking) and socioeconomic variables, *x*_*k*_, to the drug use status of individuals such as:

3 $$ y=\alpha +\sum \limits_k{\beta}_k{x}_k+\varepsilon $$

where *β*_*k*_ are marginal effects obtained from the logistic regression. The *C*_*n*_ for drug use can be decomposed as:

4 $$ {C}_n=\frac{\sum \limits_K\left(\frac{\beta_K{\overline{X}}_K}{\mu}\right){C}_K}{1-\mu }+\frac{\frac{GC_{\varepsilon }}{\mu }}{1-\mu } $$

Where ${\overline{x}}_k$ is mean of each of the independent variables, *C*_*k*_ is the concentration index for the explanatory variable *x*_*k*_, and *GC*_*ε*_ is the generalized C for *ε*. The first part of equation ((4) indicates the contribution of each explanatory factors to the overall *C*_*n*_. Based on equation (4, the *C*_*n*_ can be decomposed into two components. The first component, $\sum \limits_k\left(\frac{\beta_k{\overline{x}}_k}{\mu}\right){C}_k/1-\mu$, indicated how the *C*_*n*_ is explained by the relationship between the independent variable and explanatory variables and the systematic changes of independent variables in the distribution of socioeconomic groups. The negative (positive) contribution of independent variable to the *C*_*n*_ indicated that the distribution of independent variables across SES and the relationship of this variable to drug use are likely to contribute to the higher (lower) concentration of drug use among the poor. The second component or *GC*_*ε*_/*μ*/1 − *μ* indicated the inequality that is not explained by independent variables included in the model. Multivariable logistic regressions were then used to examine adjusted odds ratios (OR).

### Ethical statement

While each cohort center received the ethical approval from local universities, for the purpose of this study and pooling all PERSIAN data, the ethics committee of Kermanshah University of Medical Sciences approved the study (IR.KUMS.REC.1397.187).

## Results

The mean age of participants was 49.7 years [SD: 9.2]. Approximately55.5% of the participants were women. 90.9 and 20.2% of participants were married, and illiterate, respectively. In addition, 14% of the participants in PERSIAN cohort were a current smoker. 11.9% (24.1% among men versus 2.2% among women) of the population had a history of drug use.

Table 1 reported the prevalence of drug use among adults in Iran by the respondents’ characteristics in the PERSIAN cohort. As the prevalence of drug use linearly increased by age in women but did not change significantly in men (*P* = 0.7). The highest prevalence of drug use was in married people. The prevalence of drug use was decreased by education level.

**Table 1:** Distribution of the demographic characteristics among the participants and the prevalence of drug use in Iran by the respondents’ characteristics in the PERSIAN cohort

Variables		N (%)	Total prevalence	Male prevalence	Female prevalence
**Total**		**130,570 (100)**	**11.9 (15600)**	**24.1 (13998)**	**2.2 (1602)**
**Age group**	35–40	27,005 (20.7)	9.3	20.6	1.1
	41–45	24,212 (24.2)	11.7	25.0	1.5
	46–50	22,645 (22.6)	12.5	26.2	1.8
	51–55	20,297 (15.5)	12.8	25.2	2.7
	55–60	17,570 (13.5)	13.5	25.3	3.4
	61–65	12,271 (9.4)	13.1	23.7	3.8
	> 66	6570 (5.0)	12.4	21.1	4.2
**Marital status**	Married	118,769 (91.0)	12.6	23.9	2.0
	Single	2895 (2.2)	6.1	27.4	0.4
	Divorced/widowed	8906 (6.8)	5.7	34.3	4.1
**Years of education**	Illiterate (0 year)	26,666 (20.2)	8.3	23.6	3.2
	1–5 years	41,975 (31.9)	11.2	26.7	2.3
	6–9 years	23,938 (18.2)	17.9	30.3	2.0
	10–12 years	22,552 (17.1)	14.4	24.2	1.2
	> = 13 years	16,659 (12.6)	7.6	12.0	0.6
**Cigarette smoking**	Never smoker	102,141 (78.2)	4.5	10.0	2.0
	Current smoker	18,331 (14.0)	43.3	44.9	12.2
	Former smoker	10,097 (7.8)	30.6	33.1	7.7
**Alcohol consumption**	No	118,772 (91.0)	8.4	17.9	2.2
	Yes	11,795 (9.0)	47.7	48.8	11.9
**Water pipe smoking**	No	115,979 (88.8)	9.2	20.2	1.7
	Yes	14,581 (11.2)	34.0	40.5	12.9
**Physical activity Daily** **(MET-hours per day)**	24–36.5	43,226 (33.1)	14.3	26.9	3.0
	36.6–44.9	61,532 (47.2)	8.3	21.8	1.8
	≥45	25,673 (19.7)	16.8	23.4	1.9
**Economic status**	1st quintile (the poorest)	26,159 (20.0)	11.7	30.0	3.1
	2nd quintile	26,148 (20.0)	12.8	27.5	2.4
	3rd quintile	26,105 (20.0)	12.2	24.9	2.1
	4th quintile	26,095 (20.0)	12.7	23.8	1.8
	5th quintile (the richest)	26,063 (20.0)	10.3	17.6	1.2

**Fig. 1:**
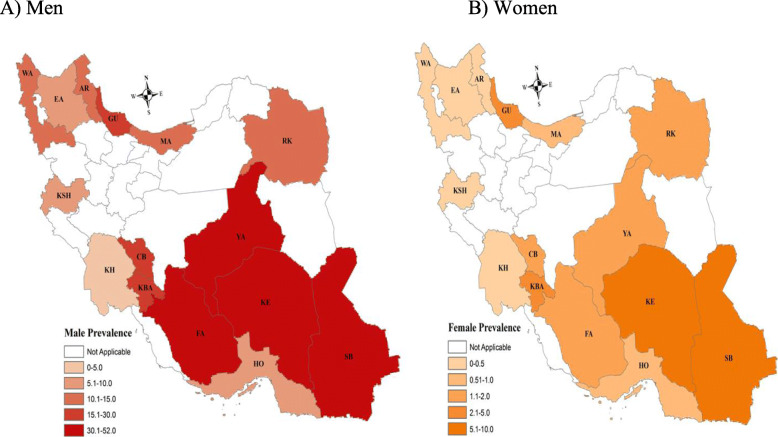
Prevalence of drug use in men and women across 15 provinces in Iran

Figure 1 shows sex difference in the prevalence of drug use in different provinces of Iran. As shown in the figure, the prevalence of drug use among men was found to be higher in Kerman (KE), Yazd (YA), Kohgiluyeh and Boyer-Ahmad (KBA), and Chaharmahal and Bakhtiari (CB) provinces. Among women, higher prevalence was found in the provinces of Sistan and Baluchestan (SB), Kerman (KR) and Gilan (GU).

**Fig. 2:**
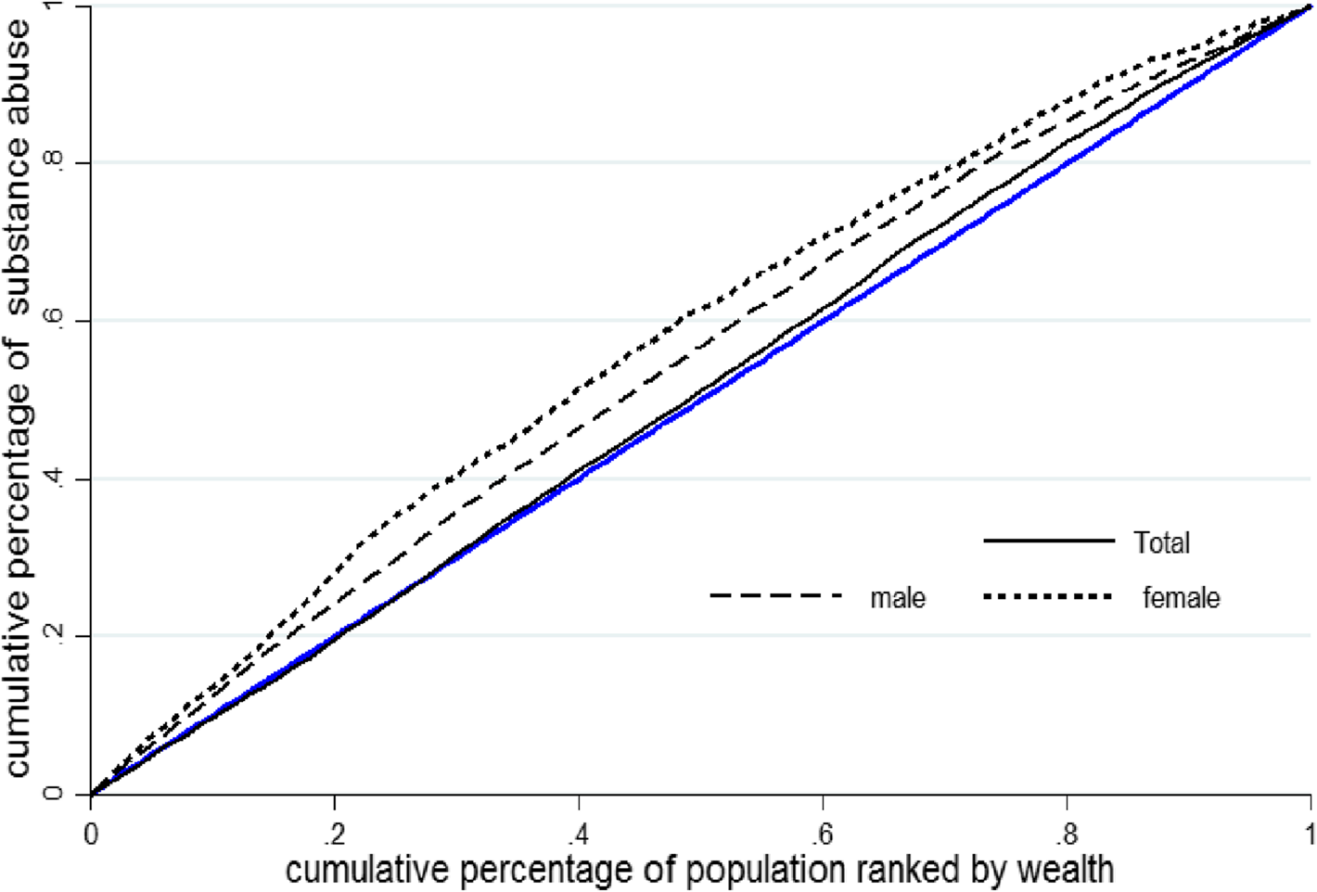
Concentration curves for drug use among men and women in Iran

The estimated C was equal to − 0.021, suggesting the higher concentration of drug use among the population with low SES. Moreover, socioeconomic inequality in drug use was found to be higher in women than in men. This is also evident in Fig. 2 where the Concentration curve for drug use for female lies above to the Concentration curve for male. The 45° line (blue line) is the perfect inequality that is equal to average health status.

**Fig. 3:**
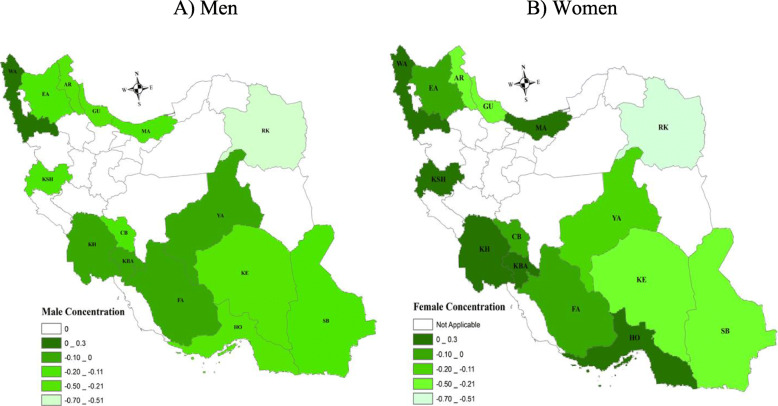
Socioeconomic inequalities in drug use in men and women across 15 provinces in Iran

All provinces, except for Khouzestan (KH) and West Azarbaijan (WA), the estimated value of *C*_*n*_ showed that druguse is concentrated among low SES groups. The sign of the *C*_*n*_ was positive for women in the provinces such as Kermanshah (KSH), Mazandaran (MA), Khouzestan (KH), Hormozgan (HO), West Azerbaijan (WA) and Kohgiluyeh and Boyer-Ahmad (KB). For men, the *C*_*n*_ value was positive for Khuzestan (KH) and West Azerbaijan provinces only. The pattern of socioeconomic-related inequality for men in the cohort population was similar to women (see Fig. 3).

### Determinants of socioeconomic-related inequalities

Due to the Simpson paradox [22] on the total value of the *C*_*n*_, decomposition analysis was performed for men and women, separately. Simpson paradox or reversal paradox is a phenomenon in probability and statistics, in which a trend appears in several different groups of data but disappears or reverses when these groups are combined. Table 2 presents the results of the decomposition of the *C*_*n*_. The results of *C*_*k*_ revealed that the prevalence of variables like being single, widowed/divorced, underweight, older ages, smoking and higher levels of physical activity is more concentrated among low SES groups. In contrast, the prevalence of alcohol consumption, obesity and overweight is higher in the high SES groups. The values of *C*_*k*_ indicated that higher concentrations of obese and overweight individuals among high-SES.

**Table 2:** The results of the decomposition analysis of socioeconomic inequality in drug use for men and women in Iran

Variables		Men						Women					
		adjusted OR	Elasticity	Concentration Index (C_**k**_)	Absolute Contribution	Percentage contribution	Summed Percentage contribution	Adjusted OR	Elasticity	Concentration Index (C_**k**_)	Absolute Contribution	Percentage contribution	Summed percentage contribution
**Age group** (Ref: 35–40)	41–45	0.65 (0.38_1.9)	0.0397	0.0549	0.0029	−2.1394	−0.42	3.58 (0.54_10.31)	0.0127	0.0953	0.0012	−0.7068	9.76
	46–50	1.10 (0.81_1.49)	0.0524	0.0581	0.0040	−2.9949		3.24 (0.48_4.49)	0.0191	0.0424	0.0008	− 0.4737	
	51–55	1.09(0.94_1.26)	0.0414	0.0258	0.0014	−1.0482		1/17(0.30_7.28)	0.0299	−0.0382	−0.0012	0.6673	
	55–60	1.15(0.91_1.44)	0.0309	−0.0766	−0.0031	2.3274		1.51 (0.17_5.9)	0.0314	−0.1555	−0.0050	2.8559	
	61–65	1.28 (1.07_1.51)	0.0141	− 0.1681	− 0.0031	2.3376		0.24 (0.29_1.96)	0.0250	−0.2841	− 0.0073	4.1537	
	> 66	1.09(0.94_1.26)	0.0037	−0.3014	−0.0015	1.1017		2.70 (1.12_6.42)	0.0135	−0.3869	−0.0053	3.0459	
**Marital status** (Ref: Married)	Single	0.73 (0.49_1.07)	−0.0007	−0.0680	0.0001	−0.0473	0.17	0.21 (0.12_0.39)	−0.0060	−0.0687	0.0004	−0.2410	0.25
	Divorce/widowed	1.53 (1.02_2.28)	0.0015	−0.1499	−0.0003	0.2171		1.64 (1.58_2.03)	0.0032	−0.2633	−0.0008	0.4860	
**Smoking status** (Ref: Never smoker)	current smoker	5.75 (5.10_6.47)	0.3271	−0.0875	−0.0377	28.1203	32.04	0.28 (0.22_0.38)	0.0160	−0.3203	−0.0052	2.9986	4.34
	Former smoker	3.92 (3.41_4.50)	0.0932	−0.0428	− 0.0053	3.9201		1.69 (1.24_2.31)	0.0060	−0.3571	−0.0022	1.2429	
**Alcohol consumption** (Ref: No)	Yes	6.97 (2.39_20.2)	0.1444	0.0872	0.0166	−12.3680	−12.37	8.80 (7.80_9.92)	0.0023	0.4644	0.0011	−0.6310	−0.65
**water pipe Smoking** (Ref: No)	Yes	1.58 (1.42_1.74)	0.0717	0.1209	0.0114	−8.5231	−8.52	2.27 (1.61_3.21)	0.0173	0.0683	0.0012	−0.6924	−0.71
**Physical activity Daily METs** (Ref: 24–36.5)	36.6–44.9	0.83 (0.74_0.93)	−0.0335	0.0776	−0.0034	2.5546	−0.80	0.63 (0.56_0.69)	−0.0305	0.0575	−0.0018	1.0260	1.17
	≥45	0.77 (0.68_0.87)	−0.0175	−0.1955	0.0045	−3.3585		0.60 (0.50_0.72)	0.0010	−0.1866	−0.0002	0.1144	
**Socioeconomic status** (Ref: 1st quintile (the poorest)	2nd quintile	0.76 (0.62_0.91)	−0.0122	−0.5276	0.0085	−6.3293	80.04	0.78 (0.64_0.84)	−0.0186	−0.2965	0.0056	−3.2268	26.37
	3rd quintile	0.63 (0.52_0.76)	−0.0225	−0.1407	0.0042	−3.1165		0.69 (0.55_0.74)	−0.0235	0.1149	−0.0028	1.5769	
	4th quintile	0.53 (0.43_0.64)	−0.0408	0.2804	−0.0151	11.2458		0.61 (0.41_0.66)	−0.0271	0.4974	−0.0138	7.8742	
	5thquintile (the richest)	0.31 (0.25_0.37)	−0.1063	0.7491	−0.1048	78.2428		0.39 (0.30_0.45)	−0.0399	0.8394	−0.0342	19.5650	
**Province** (Ref: Kermanshah (KSH))	Guilan (GU)	1.14 (0.91_1.41)	0.0258	−0.1858	−0.0063	4.7054	−0.97	24.09 (16.8_28.9)	0.1341	−0.1373	−0.0188	10.7623	−22.38
	Fars (FA)	7.46 (5.91_9.41)	0.2125	−0.3701	−0.1035	77.2690		12.18 (6.01_24.6)	0.0711	−0.3848	−0.0280	15.9918	
	East Azerbaijan (EA)	0.39 (0.31_0.49)	−0.0084	−0.0375	0.0004	−0.3086		0.40 (0.13_1.22)	−0.0034	0.0124	0.0000	0.0247	
	Mazandaran (MA)	1.32 (1.02_1.70)	0.0232	0.1624	0.0050	−3.7020		4.36 (2.03_9.31)	0.0155	0.1544	0.0025	−1.4028	
	Sistan and Balouchestan (SB)	6.44 (4.26_9.71)	0.0847	0.0507	0.0057	−4.2201		17.15 (13.39_19.24)	0.1693	0.0303	0.0052	−2.9998	
	Yazd (YA)	5.85 (4.53_7.55)	0.0827	0.1501	0.0164	−12.2018		7.92 (3.77_14.24)	0.0269	0.1959	0.0054	−3.0760	
	Kerman (KE)	7.91 (6.26_9.97)	0.1505	0.2663	0.0528	−39.3871		19.21 (16.62_24.39)	0.1840	0.3169	0.0596	−34.1030	
	Khouzestan (KH)	2.03 (1.32_3.09)	−0.0013	−0.1443	0.0002	−0.1781		0.49 (0.14_1.61)	−0.0025	− 0.2012	0.0005	− 0.2906	
	Chaharmahal and Bakhtiari (CB)	2.62 (2.07_3.30)	0.0328	0.4504	0.0194	−14.5110		10.01 (7.51_13.91)	0.0224	0.4702	0.0108	−6.1554	
	Hormozgan (HO)	0.96 (0.87_1.12)	0.0019	−0.0463	−0.0001	0.0865		4.02 (1.6_9.85)	0.0027	−0.0538	−0.0001	0.0838	
	West Azerbaijan (WA)	1.06 (0.62_1.46)	0.0034	−0.1115	−0.0005	0.3721		2.47 (0.92_6.53)	0.0020	−0.1677	−0.0003	0.2001	
	Ardabil (AR)	0.85 (0.73_1.51)	0.0086	0.2446	0.0028	−2.0755		1.89 (0.78_4.55)	0.0038	0.2240	0.0009	−0.4950	
	RazaviKhorasan (RK)	1.77 (0.64_1.12)	0.0110	0.5949	0.0086	−6.4323		11.82 (9.2_13.90)	0.0229	0.6878	0.0161	−9.2199	
	Kohgiluyeh and Boyer-Ahmad (KB)	0.65 (0.80_3.90)	0.0016	0.2434	0.0005	−0.3888		16.93 (14.1_18.29)	0.0024	0.2488	0.0006	−0.3454	
***Summed***					**−0.1193**		**89.17**				**−0.0151**		**18.15**
***Residual***					**−0.0147**		**10.83**				**0.1559**		**81.85**
The C_n_					**−0.1340**		**100.00**				**0.171**	**−0.1710**	**100.00**

For women, SES (26.37%), residence province (− 22.38%) and age (9.76%) had the most significant contribution to socioeconomic inequality in drug use, respectively. For men, SES (80.04%), smoking (32.04%) and alcohol consumption (− 12.37%) were the main contributors to socioeconomic inequality in drug use in Iran.

## Discussion

Our study indicated that 11.9% (24.1% among men versus 2.2% among women) of the adult population in Iran used the illicit drug more than one time in their lifetime. In addition, 14 and 9% of the participants were a current smoker and had history of alcohol consumption, respectively. These results are in line with the findings of earlier studies investigating the drug use, cigarette smoking and alcohol consumption among Iranian adults. For example, the obtained results of study carried out by Jalilian et al. [15] indicated that 19.4 and 10.1% of the Iranian adults reported history of cigarette smoking, and alcohol drinking respectively. As well as, Mohebbi et al. [16] carried out a study on 2065 adults aged 18 years and older in Iran and reported that lifetime experience of drug use was 12.9% (21.5% among men versus 4% among women).

Drug use is a concerning health problem among Iranian population [23]. Moreover, since the alcohol drink and narcotics are illegal in Iran we thought that fear of confrontation with legal authorities may affect our study participation rate [24]. These results can be warning to health policy makers in Iran.

Our findings suggested that drug use is more concentrated among the population with low socioeconomic status in Iran. This result is similar to the results reported by other studies. For example, Lawana and Booysen in their study in South African reported alcohol use is more concentrated among the lower socioeconomic groups [25]. Furthermore, Nikfarjam et al. in their study in total 12,293 samples in Iran reported the relative frequency of alcohol use among males was about 8 times higher than females [26]. Evidence points to the lower socio-economic groups suffer multiple deprivations [27]. For example, studies have been indicated association between lower SES and the following risk factors: cigarettes smoking; drug use problems; psychosocial stress; obesity; lack of social support; and less use of health care services [27–29]. Our study indicated that the drug use is more concentrated among the lower SES groups. Concerted government efforts, within the health sector (for example, pay special attention to groups with lower SES for drug addiction treatment) is required for eliminate inequality.

Furthermore, SES.

For men, SES (80.04%), smoking (32.04%) and alcohol consumption (− 12.37%) were the main contributors to socioeconomic inequality in drug use.

Also, the results of our study indicated that the SES had more contribution to socioeconomic inequality in drug use among men compared to women (80.04% VS 26.37%). Moreover, drug use prevalence was higher among the male population (24.1% among men versus 2.2% among women). This finding is in line with other studies that indicated that men are at higher risk of drug use than women [11, 30, 31]. A study by Do et al also indicated a higher proportion of drug use among men as compared than women in Vietnam [14]. Thus, specific strategies in order to prevention of drug use for this group should be dispensed by policy-makers.

Another finding of current study was indicated the prevalence of drug use was decreased by increased education level. In this regard, Lee et al. in their longitudinal study on 808 male and females elementary school students followed to age 30 indicated earning a high school diploma lessens the risk of drug use problems which contribute to economic instability in young adulthood [28]. Also, in line with our findings Kessler et al. carried out a study on 9282 English speakers 18 years and older in the coterminous United States and reported that lower education was associated with increased comorbid mental health and drug use problems [32]. Several studies have underlined higher education level as a protective factor against drug use [16, 33]. Our findings suggest that the drug use prevention program should focus on less-educated adults in Iran.

Our study also showed that the prevalence of drug use linearly increased by age in women but did not change significantly in men. Moreover, the highest prevalence of drug use was in married group. These findings are somewhat consistent with other studies in Iran. For example, Amin-Esmaeili et al. reported that the odds of drug use related-disorders were higher in previously married as compared to currently married or single adults in Iran [34]. However, Mohebbi et al. demonstrated that divorced or widowed adults reported a higher rate of opium use which is inconsistent with the present study found [16]. It is also inconsistent with our findings, Motevalian et al. in their study indicated that the prevalence of drug use increased in men and women with an increase in age [35].

Because of proximity to Afghanistan (the country with the greatest opium production in the world) Iran seems to have the conditions for being a significant place for drug use [4]. As our findings show, drug use was more prevalent in neighboring provinces with Afghanistan.

### Study limitations

Although our study has several strengths including large sample size, the results reported in this study should be interpreted in light of some limitations. First, our study is cross-sectional nature; thus, no causal inference can be derived from the associations reported in the paper. Second, self-reporting information may be subject to recall bias. Third, since the inclusion age of our study was 35 years and older, it may affect one affect the results and the estimated lifetime prevalence and type of drug use. Finally, the our study investigated drug use and alcohol consumption history more than one time during lifetime using yes-no scale, which was the main limitations of the current study and asks for more attention.

## Conclusions

There are multiple factors to explain the drug use inequality among Iranian people. The present study confirmed the applicability of the SES to explain drug use among Iranian population. We found that higher rate of drug use among low-SES. Thus, drug use prevention programs in Iran should target low SES adults in order to reduce inequalities in drug use in Iran. Our finding could be useful for health policy maker to design and implement effective preventative programs to protect Iranian population against the drug use.

## Data Availability

Data used for this study can be accessed upon request from the first author (Dr. Moradinazar) at m.moradinazar@gmail.com

## Appendix

**Table 3:** The cohort population in the study provinces

Row	Province	*Population	Cohort site	*Population	Cohort population	Main Ethnicities
1	Ardabil	1,270,420	Ardabil	529,374	8192	Azeri (Turk)
2	Chaharmahal and Bakhtiari	947,763	Sharekord	93,104	6664	Lur
3	East Azerbaijan	3,909,652	Khameneh	3056	14,978	Azeri (Turk)
4	Fars	4,851,274	Kavar	31,711	2244	Fars (Persian), Turk
			Kharameh	18,477	10,662	Fars (Persian), Arab
			Fasa	110,825	10,113	Fars (Persian), Arab and Turk
5	Guilan	2,530,696	Some’e Sara	58,658	10,512	Gilaki
6	Hormozgan	1,776,415	Bandare Kong	19,213	3570	Arab
7	Kerman	3,164,718	Rafsanjan	161,909	9982	Fars (Persian)
8	Kermanshah	1,952,434	Ravansar	47,657	10,077	Kurd
9	Khouzestan	4,710,506	Hoveizeh	19,481	9156	Arab
10	Mazandaran	3,283,582	Sari	309,820	10,253	Tabari
11	RazaviKhorasan	6,434,501	Mashhad	3,001,184	2189	Fars (Persian)
			Sabzevar	243,700	784	Fars (Persian)
12	Sistan and Balouchestan	2,775,014	Zahedan	587,730	8318	Balouch
13	West Azerbaijan	3,265,219	Ghoushchi	2787	3662	Azeri (Turk)
14	Yazd	1,138,533	Shahedieh, Yazd	18,309	9901	Fars (Persian)
15	Kohgiluyeh and Boyer-Ahmad	7,313,052	Dena	52,242	421	Lur

*The frequency of population is according to Iranian Population and Housing Census in 2016

## Abbreviations

C: Concentration Index
KUMS: Kermanshah University of Medical Science
MoHME: Ministry of Health and Medical Education
NHIS: National Health Interview Survey
NCDs: Non-communicable Diseases
OR: Odds Ratios
PCA: Principal Component Analysis
PERSIAN: Prospective Epidemiologic Research Study in Iran
SES: Socioeconomic Status
WHO: World Health Organization
